# Textural Analysis as a Predictive Biomarker in Rectal Cancer

**DOI:** 10.7759/cureus.32241

**Published:** 2022-12-06

**Authors:** Mahmoud Alrahawy, Medhet Aker, Mohmed Issa, Omer Ali, Khaled Noureldin, Ahmed Gaber, Ahmed Mahgoub, Mohamed Ahmed, Mahmoud Yousif, Ashraf Zeinaldine

**Affiliations:** 1 General Surgery, Menoufia University, Menoufia, EGY; 2 General and Colorectal Surgery, Colchester Hospital University, Colchester, GBR; 3 General Surgery, Russells Hall Hospital, Birmingham, GBR; 4 Surgical Oncology, Royal Blackburn Hospital, Lancashire, GBR; 5 General Surgery, Cairo University Hospital, Cairo, EGY; 6 Colorectal Surgery, Southend University Hospital NHS Foundation Trust, Essex, GBR; 7 Surgery, Frimley Park Hospital, Surrey, GBR; 8 Surgery, James Cook University Hospital, Middlesbrough, GBR

**Keywords:** recurrence predictors, life prognosis, imaging biomarker, : rectal cancer, textural analysis

## Abstract

Colorectal cancer (CRC) is a common deadly cancer. Early detection and accurate staging of CRC enhance good prognosis and better treatment outcomes. Rectal cancer staging is the cornerstone for selecting the best treatment approach. The standard gold method for rectal cancer staging is pelvic MRI. After staging, combining surgery and chemoradiation is the standard management aiming for a curative outcome.

Textural analysis (TA) is a radiomic process that quantifies lesions’ heterogenicity by measuring pixel distribution in digital imaging. MRI textural analysis (MRTA) of rectal cancer images is growing in current literature as a future predictor of outcomes of rectal cancer management, such as pathological response to neoadjuvant chemoradiotherapy (NCRT), survival, and tumour recurrence. MRTA techniques could validate alternative approaches in rectal cancer treatment, such as the wait-and-watch (W&W) approach in pathologically complete responders (pCR) following NCRT. We consider this a significant step towards implementing precision management in rectal cancer.

In this narrative review, we summarize the current knowledge regarding the potential role of TA in rectal cancer management in predicting the prognosis and clinical outcomes, as well as aim to delineate the challenges which obstruct the implementing of this new modality in clinical practice.

## Introduction and background

The primary management of rectal cancer includes neoadjuvant chemoradiotherapy (NCRT) combined with surgery for remedial outcomes. Early detection of colorectal cancer (CRC) provides better oncological outcomes; therefore, accurate diagnostic and prognostic biomarkers are vital for better CRC, especially rectal cancer, which spreads more locally and presents in an advanced stage [1]. MRI is regarded as the gold standard technology for staging rectal cancer because of its ability to reveal poor prognostic characteristics such as invasion of the circumferential resection margin (CRM) and extra-mural venous invasion (EMVI). However, quantifying MRI images through new imaging analysis tools may build validated imaging biomarkers (IBMs) for outcomes prediction and treatment personalisation [2].

Texture analysis (TA) modality has evolved as a part of the radiomic field to quantify image characteristics. TA process includes statistically measuring pixels (grey-level) distribution in a selected area (region) in the medical image and translating its actual appearance (e.g. heterogenic) [3]. This narrative review encapsulates the present rectal cancer management, the concept of TA and its ability for predicting rectal cancer outcomes, and the limitations of using TA as a predictive imaging biomarker in clinical practice.

In the search strategy, we used several search terms/phrases including TA, rectal cancer, survival, locally advanced rectal cancer (LARC), tumour recurrence, distant metastasis, local recurrence, precision medicine, MRI, CT, and PET. Databases used were (Medline, Embase, and PubMed) through Healthcare Databases Advanced Search (HIDAS) service. Filters were limited to English articles and also studies on humans. After omitting duplications, abstracts were printed for the saved results. Then, these abstracts were reviewed to exclude irrelevant studies, such as non-English studies. We analysed the full text of each study for relevant information. All included articles were carefully read and the key findings along with future research suggestions were highlighted in the review to delineate the current gap in using TA in clinical settings.

## Review

Rectal cancer

Epidemiology and Diagnosis

Because of western diet and lifestyle, rectal cancer has become predominant in western countries, accounting for 30% of all colorectal tumours. In those aged younger than 65 years, the rectum is the most common site of CRC [4]. Accurate preoperative staging of rectal cancer is fundamental for selecting patients for neoadjuvant therapy (NCRT) and subsequent surgical approach to optimise management and prognosis. The Tumor, Node, Metastasis (TNM) staging system of the American Joint Committee on Cancer (AJCC) is the preferred staging system for CRC. Use of the older Astler-Coller modification of the Dukes’ classification is discouraged. Table 1 demonstrates the AJCC TNM staging classification of rectal cancer [5].

**Table 1:** AJCC TNM classification of rectal cancer AJCC: American Joint Committee on Cancer; TNM: tumor, node, metastasis.

Category	Descriptor
T category	
Tx	Primary tumor cannot be assessed or depth of penetration not specified
T0	No evidence of a primary tumor
Tis	Carcinoma in situ: intraepithelial or invasion of the lamina propria
T1	Tumor invades Submucosa
T2	Tumor invades Muscularis propria
T3	Tumor invades into the subserosa or into non-peritonealized pericolic or perirectal tissue
	A: <1 mm, B: 1–5 mm, C: 5–15 mm & D: >15 mm
T4	A: Invades the visceral peritoneum surface B: Invades surrounding organs
N category	
Nx	Regional lymph nodes cannot be assessed
N0	No regional lymph node metastasis
N1	
	A: 1 pericolic or perirectal lymph node
	B: 2–3 lymph nodes
	C: Tumor deposit(s) in the subserosa, mesentery, or nonperitonealized perirectal tissues
N2	
	A: 4–6 lymph nodes
	B: 7 or more regional lymph nodes
M category	
M0	No distant metastasis
M1	Distant metastasis

Rectal cancer workup starts with history taking and physical examination (including digital rectal examination) combined with routine investigations (e.g. blood count, liver, and renal functions) and serum carcinoembryonic antigen (CEA). Endoscopy with a biopsy is vital for histopathological confirmation. Faecal blood tests could detect asymptomatic bleeding tumours, such as the guaiac faecal occult blood test (gFOBT) and the faecal immunochemical test (FIT). Colonoscopy is considered the gold standard for CRC screening and can offer both diagnosis and therapy [6].

Computed tomography (CT) ideally detects pretreatment distant metastases (e.g. liver). Still, it is not utilised in stratifying the patients with rectal cancer for a tailored treatment regimen because of its suboptimal assessment of local T stage and N status in rectal cancer. MRI is the standard technique used for TNM staging in rectal cancer because of its high ability to assess poor prognostic markers such as nodal disease (ND), CRM invasion, and EMVI, making it a viable alternative to endo-rectal ultrasound (ERUS) [7]. MRI’s role as a predictive imaging biomarker of rectal cancer outcomes (e.g. response to NCRT distant spread and survival) is still developing.

NCRT

NCRT, radiotherapy alone or in combination with chemotherapy, is recommended for all newly diagnosed locally advanced rectal adenocarcinoma (LARC) with a clinical (c) stage T3 or T4. The two current standards of preoperative radiation therapy are given to reduce the risk of local recurrence, namely short and long courses. Short-course radiation (SCRT) refers to 25Gy in five days, typically followed by surgery about one week after completion; meanwhile, long-course radiation (LCRT) refers to 50-54Gy in 28-30 fractions with concurrent fluoropyrimidine chemotherapy. SCRT provides a shorter treatment period and costs savings associated with this approach, but is associated with a lack of tumour downstaging before surgery [8].

Management After NCRT

Rectal cancer is curable after surgical excision. Depending on the location and severity of the condition, different surgical procedures may be used, aiming to achieve total mesorectal excision (TME), may be used [9]. Although the patients achieving complete pathological response represent one-fifth of rectal cancer patients following neoadjuvant chemoradiation, there is no current level I evidence to support a wait-and-watch (W&W) approach in complete responder after NCRT in rectal adenocarcinoma. Since patients with pCR have excellent oncological outcomes, there has been a booming interest in organ preservation W&W for those who develop a pCR [10]. Therefore, further research on improving predicting modalities that accurately assess LARC post-treatment response is mandatory for more accurate predictability of tumour outcomes.

TA

Introduction on Radiomics

Radiomics is a science combining radiology, mathematical modelling, and deep machine learning (DML) to analyse medical image texture and establish IBMs from different scans (CT, MRI, ultrasound, PET). IBMs can identify the unique pathological changes of different lesions with different image textures. IBMs can subsequently serve as a noninvasive “virtual biopsy” that provides unique details on cancer heterogenicity and phenotype. This technique may improve diagnosis, prognostication, and clinical decisions, to deliver tailored therapy for each patient separately [11].

**Figure 1:**
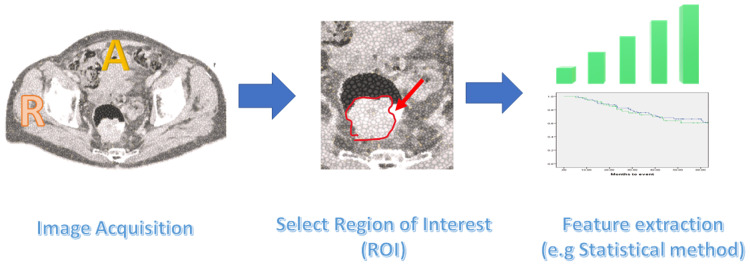
Radiomic/textural analysis process and textural feature extractions

Radiomic analysis process includes image acquisition, segmentation of the region of interest (ROI), and extraction of the radiomics features (Figure 1). The use of artificial intelligence (AI), especially machine learning (ML) approaches, improves the overall quality [12]. Correlating extracted radiomics features to the clinical outcomes could provide a step forward to personalised treatment and precision medicine.

Concept of TA

Image TA is part of radiomics and provides an objective quantitative assessment of tumour heterogeneity. It is a new imaging analysis modality that describes various techniques to measure grey-level patterns and pixel inter-relationships. It gives more visibility to different image areas that are sometimes imperceptible to the human eye. The methods used in TA of digital images vary widely. Tuceryan and Jain (1993) categorised TA methods into four types. The statistical approach to measuring the distribution of grey levels comes first. Geometrical analysis of texture primitives, a model-zased method for creating a parametric model of the intensity distribution, and signal processing for the frequency content of the image are all examples of TA [13].

In statistical method of TA, commonly used in TA field, local features (grey-level distributions) are calculated at the area of interest in the image and produce a histogram distribution of gray-level intensities from the extracted features. Pixels in the image (e.g. MRI) reflect the body content of water, and based on the quantity of pixels examined in the selected image segment, statistical TA techniques can be divided into further categories, including 1st and 2nd order statistics. First order statistics studies one pixel values distribution such as mean (average), standard deviation (SD), kurtosis (flatness or peakedness), and skewness (asymmetry). It does not include the spatial relationship, and it is measured through histogram-based analysis. Second order statistics compute the association between the chosen pixel of interest (POI) and its surrounding pixels. Entropy (which quantifies the disorder of the intensity distribution) and grey level cooccurrence matrix (GLCM), which depicts diverse combinations of grey levels within the image, are two examples of 2nd order statistics. Higher-order statistics measure the relationship amongst three or higher pixels after applying filters/mathematical tuning to the images that provide more patterns and details with a specific texture. These calculated textural values assess the degree of heterogenicity in the ROI in medical images and reflect upon the nature of the ROI (eg, lesion versus”normal”) and its potential behavior, shifting medical image interpretation from visual perception of macrotexture into quantified analysis of microtexture [14].

**Figure 2:**
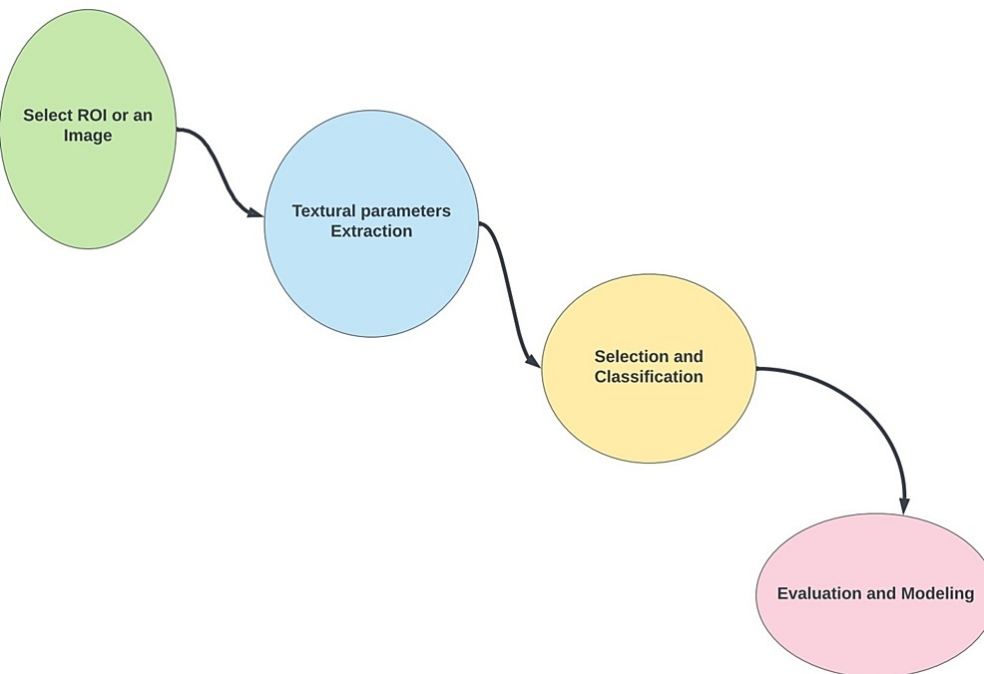
Process of texture analysis of medical images or ROI ROI: region of interest.

The flowchart outlines the multi-step process of TA (Figure 2). It includes the input steps such as image acquisition and reconstruction, image segmentation (selecting ROI). Then, output stage, which includes the extraction of textural features (e.g. shape features; first-, second-, and higher-order features), classification of features, analysis (e.g cluster analysis, neural network, linear/logistic regression, and others) and eventually construction of a model that could be generalised in clinical practice. These radiomic models could be used in clinical settings for predicting prognosis, non-invasive disease monitoring, and assessment of the effectiveness of treatment [15].

TA in Medical Imaging

Recent studies have indicated a correlation between CT texture features tumours characteristics that reflect tumour grade, cellular processes (e.g. hypoxia or angiogenesis) and genetic markers such as KRAS or epidermal growth factor receptor (EGFR) mutation status. Therefore, CT Textural analysis (CTTA) can differentiate benign from malignant or biologically aggressive lesions. CTTA showed promise in predicting prognosis and treatment response in CRC, head and neck cancer, oesophageal cancer, lung cancer, and renal cell carcinoma (RCC) [16].

MRTA also can assess tumour heterogeneity and predict clinical outcomes such as survival and treatment response in different kinds of cancers (e.g cervical and breast cancers cancer) [17]. In locally advanced rectal cancer patients, MRTA also identified pathologically complete responders (pCR) after their NCRT [18]. This technique can be implemented in cancer precision medicine and prognosis monitoring.

TA and Rectal Cancer Outcomes

Some studies established a correlation between TA and rectal cancer outcomes such as treatment response, survival, and distant spread [19-21].

LARC management starts with downstaging chemoradiotherapy. TA was examined recently for predicting the pathological response of NCRT in LARC patients. One research showed that complete responders to NCRT in LARC have different textural properties (e.g. mean, entropy, skewness, and mean positive values) than partial responders whose TA did not show the same features [18]. In another study, textural parameters extracted from MRI scans before and after NACRT marked the patients with complete responses to neoadjuvant treatment. Kurtosis was able to differentiate complete responders from partial responders or non-responders. It was higher in the midtreatment MRI scans in patients with pCR but lower in the pretreatment ones [22].

Survival analysis using textural parameters and represented promising results. A TA study highlighted that textural features extracted from pre-and post-treatment MRIs were significant markers of overall survival (OS), disease-free survival (DFS) and relapse-free survival (RFS) in LARC patients. OS was predicted by both pretreatment MPP and post-treatment skewness, meanwhile, from texture features extracted from post-treatment scans, entropy and kurtosis, extracted from post-treatment scans, predicted DFS and RFS [23].

MR morphological and textural features from the baseline rectal MRI could be valuable for predicting tumour recurrence in LARC patients. First order and second order (higher statistical) textural features can define either local or distant tumour recurrence according to recent research work [24].

Ganeshan et al. [25] depicted a correlation between TA and the prediction of liver metastasis in CRC. They assessed whether CTTA was able to predict patients who eventually developed hepatic metastasis in a CRC study. Results showed that entropy and uniformity, were significant predictors of patients’ liver metastases in comparison to patients with normal liver. This explores the potential role of TA in predicting distant spread of rectal cancer that may personalize the overall management considering specific neoadjuvant protocol to decrease changes of metastasis from the start of therapy.

TA and Other Cancers

TA has shown an ability to predict oncological outcomes in other types of cancer. MRI textural features predict the recurrence of advanced cervical cancer treated with concurrent chemoradiotherapy (CCR). RunLengthNonuniformity (RLN) from T2 and energy are two MRI predictors of cervical cancer recurrence in recent research [26]. Other studies also showed a correlation between TA and oncological outcomes (e.g survival and neoadjuvant response) in the lung, esophageal head and neck, and renal cancers as well [27]. Table 2 summarizes the reviewed studies with the key findings on the ability of TA to predict clinical outcomes.

**Table 2:** Some studies on textural analysis as a potential imaging biomarker TA: textural analysis; MRTA: MRI textural analysis; pCR: pathologically complete responders; LARC: locally advanced rectal cancer; NCRT: neoadjuvant radiotherapy; CTTA: CT textural analysis; CRC: colorectal cancer; ADC: apparent diffusion coefficient; fp-AML: fat-poor angiomyolipoma; RCC: renal cell carcinoma.

Author/Date	Question	Method/sample	Findings	Future Research
Aker et al. (2019) [18]	Can TA of MRI (MRTA) work as a quantitative imaging biomarker to accurately identify LARC patients with` pCR?	Retrospective diagnostic accuracy study/114 LARC patients after NCRT	5 TA parameters (Mean, SD, entropy, mean of positive pixels, and skewness) were all able to identify CR after NCRT.	Further studies to standardise method (e.g. TA features extraction, scans timing, and acquisition parameters)
Devoto et al. (2022) [21]	Can radiomics analysis assess treatment response after neo-adjuvant therapy?	Retrospective / 114 patients with rectal cancer who underwent magnetic resonance (MR) imaging after NCRT	T2-weighted-based radiomics classified pCR patients better than qualitative assessment	For clinical translation, Validation on a larger data set is required
de Cecco et al. (2015) [22]	Whether MRI texture features of rectal cancer can predict tumoral response after NCRT?	Prospective/ 15 consecutive patients	T2w MR Texture parameters (e.g. kurtosis) derived from have the potential to act as imaging biomarkers of tumoral response after NCRT	
Jalil et al. (2017) [23]	Whether MRI textural features of rectal cancer patients can predict long-term survival after long-course chemoradiotherapy?	A retrospective cohort/56 stage 2 &3 rectal cancer	MR based TA of rectal cancers (e.g. mean, mean positive pixels) can predict rectal cancer patients’ outcome before surgery (and select patients for individualized therapy).	Validation on histological correlations of rectal cancer heterogeneity for different MR texture scales.
Park et al. (2020) [24]	Can imaging features and texture analysis (TA) based on baseline rectal MRI predict therapeutic response to NCRT and tumour recurrence in LARC?	Retrospective cohort/ 78 LARC patients	In LARC, imaging features and MRTA could predict treatment response to NCRT for and tumour recurrence.	Validation and identification of consistently valuable features for various applications.
Ganeshan et al. (2009) [25]	whether texture analysis of non-contrast enhanced computed tomography (CTTA) images in apparently disease-free areas of the liver is altered by extra- and intra-hepatic malignancy in colorectal cancer patients ?	Case-control/31 patients	CTTA unenhanced hepatic CT scan depicts changes in apparently normal liver (in patients with CRC malignancy) compared to normal patients.	
Meng et al. (2018) [26]	Can MRI features , from T2WI and apparent diffusion coefficient (ADC) maps, predict the recurrence of advanced cervical cancer patients treated with concurrent chemoradiotherapy (CCRT)?	Prospective/ 34 advanced cervical cancer patients	T2 and ADC textural parameters as non-invasive imaging biomarkers in early predicting recurrence in advanced cervical cancer treated with CCRT,	- Larger sample size and an external validation. - Longitudinal study for long-term prognostic value of MRTA in cervical cancer.
Zhang et al. (2020) [16]	Can CTTA differentiate between fat-poor angiomyolipoma (fp-AML) from renal cell carcinoma (RCC)?	systematic review /290 studies were included	CT texture features such as entropy () is a potential noninvasive, imaging biomarkers that differentiated between fp-AML from RCC on both unenhanced CT and enhanced CT.	Due to lack of standards and reproducibility, future effort to standardise the definitions and process. Prospective studies to avoid bias

All the above-mentioned knowledge can prove the ability of TA of medical images to be used as a potential imaging biomarker for risk stratification and prognostication of rectal cancer. This could be a significant step forward in building a TA model for more precision medicine implementation in rectal cancer management.

Challenges of TA use in clinical practice

Creating a reliable TA biomarker is still challenging. Biomarker is defined as an objective sign of medical state observed from outside the patient, which is accurately and reproducibly measured [28]. Still, no universal imaging biomarker is available to use for quantitatively analyzing medical images, and subsequently, radiomics tools cannot be applied in routine clinical practice. Following are some of these challenges.

Challenges of Image Acquisition and Reconstruction

A major limitation of TA use is the variation in image acquisition protocols and different reconstruction algorithms amongst various institutions with different imaging scanners. Acquisition parameters are different between various clinical imaging techniques, such as image spatial resolution for CT and echo time for MRI, with variations also in individual patients. Some unstable features, subsequently, might deviate from the extracted variables’ values when retesting. When contrast is required in a medical imaging, both anatomical images (e.g. CT and MRI) and functional images (PET CT) have variability in the injected radiopharmaceutical activity, injection-to-image acquisition time, and bed position. These factors have significant implications on the reproducibility of extracted radiomics features [29]. A remedy for this issue may be to initially subtract these unstable parameters, affected by the acquisition and reconstruction process by integrating pretested information into the acquisition and reconstruction algorithms [30].

Challenges of Image Selection and Segmentation

As a critical step in TA process, image segmentation is challenging as several tumors’ express undefined borders. During quantitative TA analysis process, medical image is translated to numerical arrays, then textural features are calculated. Therefore, if segmentations differ, the quantitative imaging feature values will be influenced variably (according to each feature definition) [31]. Manual segmentation, a reader-dependent step, also requires a lot of effort and there is still no standardized segmentation protocol for all image techniques to follow. Selecting the ROI is variable among researchers. Some recommend the maximum cross-sectional diameter, meanwhile, others prefer the whole tumour volume to get more representative parameters to reflect upon tumor heterogenicity. Automatic segmentation through DML and selecting robust textural features that are not influenced by segmentation variability are potential solutions to overcome these limitations [26].

Challenges of Image Feature Extraction

In the radiomics process, different radiomics toolboxes result in variations in estimation of radiomics feature values. This is apparently because of the use of different processing software and feature definitions [32]. Number of input parameters is variable between current methods to quantitively calculate unique imaging features. To overcome that issue, preliminary analysis of the whole features offered by the computation tool can be performed to identify the most repeatable and reproducible values, which would then be reduced with redundancy analysis [33]. In tracking the response of treatment (e.g. rectal cancer after NCRT), the examined scan time-point (pre- or mid- or posttreatment) is not universal. Analysis of pretreatment scan reflects intrinsic tumour characteristics. Meanwhile, post-treatment scans may be affected by unstable variables such as fibrosis and oedema [34]. Building a reliable radiomic model is also difficult because of the redundancy of the extracted features that are not correlated to outcomes in clinical settings. As an initiative to tackle this issue, the radiomics community started an initiative-Imaging Biomarkers Standardization Initiative (IBSI) to standardize radiomics feature extraction using various toolboxes [35].

Future research

Constructing a reliable TA/radiomic model for applications in oncology and precision medicine is still challenging because of the absence of universal standards in image analysis. Therefore, several standardization approaches have been developed to address these issues, aiming to correct differences in acquisition and reconstruction prior to feature extraction, including robust features selection, and batch effect correction methods. Feature selection eliminates features with unwanted variation because of technical factors. Meanwhile, batch effect correction enables standardization following extraction with existing open-source tools [36]. An example of batch effect correcting tools is the ComBat, which is an imaging parameter harmonization method originally developed for genomics. It can rectify variation in imaging factors specific to imaging parameters by estimating position and scale parameters [37]. However, radiomic features are still affected by several biologic (lesion) and non-biologic (image acquisition) factors that require more advances over the current harmonization tools across centers and scanners, and the development of a unique global protocol for quantitative analysis scans using the currently available retrospective data remains a necessity [38].

New machine-learning techniques are required to select features for a specific endpoint. Complex computers are therefore needed to feed it with information from past examples and data sets, which may lead to the appropriate selection of required features [39]. Model validation requires more prospective data collection from clinical trials recruiting independent cohorts. Shared databases, and genomic and clinical data, among different institutions, will help create validation sets for different cancers to solve this issue.

Future prospective trials that integrate imaging features with biological and clinical data through bioinformatic tools are required. OMIC-tools, for instance, is a technique used to measure complete molecular profiles or biochemical group composition; (meta) genomics for DNAs, transcriptomics for RNAs, proteomics for proteins, metabolomics for small hydrophilic compounds, and lipidomics for small lipophilic compounds [40]. Linking -OMIC metadata sets with large amounts of TA data, along with clinical information, can translate more specific properties of lesions. The actual disease microenvironment would subsequently be reflected to augment the purposes of diagnosis, personalised treatment, and monitoring clinical outcomes. Radio-genomics is an emerging radiomic subfield that correlates the imaging phenotypes to the tumour genetic profile, aiming to express specific tissue/tumor features that translate the actual burden without requiring invasive tissue samples. This also needs correlation to histopathology at the beginning to get a standard dataset to provide a robust non-invasive imaging biomarker for clinical-decision making. Rectal cancer management is a typical example that would be modulated if a validated imaging biomarker is applied in clinical practice. W&W approach can be approved for complete responders to NACT when their MRI textural features predict a good prognosis of the tumour [41].

All these goals require tedious labor by multidisciplinary groups (clinicians, radiologists, scientists, and bioinformaticians) to provide global metadata sets and models that could build non-invasive IBMs for disease diagnosis, tracking outcomes, and precision management.

## Conclusions

This review shows that textural parameters extracted from medical images (e.g. CT or MRI) could predict important clinical outcomes in rectal cancer, such as treatment response after neoadjuvant chemoradiation, survival, tumour recurrence, and distant spread. This could build up a TA risk stratification model to classify rectal cancer patients according to their expected TA prognostic and oncological outcomes. Eventually, this could change rectal cancer management, according to the prognostic criteria, into more personalized treatment options (e.g. W&W vs surgery). However, the lack of a universal validated radiomic protocol obstructs the use of TA of medical images in clinical settings. More prospective and randomized trials that integrate clinical, biological, and radiological variables are mandatory to overcome TA standardization challenges.
